# Role of serum levels of intraoperative brain natriuretic peptide for predicting acute kidney injury in living donor liver transplantation

**DOI:** 10.1371/journal.pone.0209164

**Published:** 2018-12-17

**Authors:** Min Suk Chae, Hyunjoon Park, Ho Joong Choi, Misun Park, Hyun Sik Chung, Sang Hyun Hong, Chul Soo Park, Jong Ho Choi, Hyung Mook Lee

**Affiliations:** 1 Department of Anesthesiology and Pain Medicine, Seoul St. Mary’s Hospital, College of Medicine, The Catholic University of Korea, Seoul, Republic of Korea; 2 Department of Surgery, Seoul St. Mary’s Hospital, College of Medicine, The Catholic University of Korea, Seoul, Republic of Korea; 3 Department of Biostatistics, Clinical Research Coordinating Center, Catholic Medical Center, The Catholic University of Korea, Seoul, Republic of Korea; University of Cambridge, UNITED KINGDOM

## Abstract

**Background:**

Patients with end-stage liver disease frequently experience acute kidney injury (AKI) after living donor liver transplantation (LDLT). Serum levels of brain natriuretic peptide (BNP) have increasingly been accepted as a predictor of AKI in clinical settings. This study investigated the predictive role of intraoperative BNP levels in the early development of AKI after LDLT.

**Patients and methods:**

Adult patients (≥19 years old) who had undergone elective LDLT from January 2011 to December 2017 were classified into the non-AKI and AKI groups according to the Kidney Disease: Improving Global Outcomes criteria. Serum levels of BNP were measured three times in the preanhepatic, anhepatic, and neohepatic phases. Perioperative data in recipients and donors were analyzed retrospectively.

**Results:**

Sixty-one patients (22.4%) suffered from AKI immediately after LDLT. Severity according to AKI stage was as follows: 28 patients in stage 1 (10.3%), 18 patients in stage 2 (6.6%), and 15 patients in stage 3 (5.5%). In the neohepatic phase, both BNP levels and proportions of patients with high BNP levels (≥100 pg/mL) were higher in the AKI group than in the non-AKI group. Only BNP levels in the non-AKI and AKI stage 1 groups significantly decreased from the preanhepatic phase to the neohepatic phase; those in AKI stages 2 and 3 groups did not. In particular, BNP levels of all AKI stage 3 patients increased to more than 100 pg/mL, and the proportion of patients with high levels also increased significantly through the surgical phases in the AKI stage 3 group. In multivariate analyses, BNP levels in the neohepatic phase were significantly associated with early development of AKI after LDLT, as well as the total amount of packed red blood cells in transfusions and total duration of graft ischemia.

**Conclusions:**

Monitoring serum levels of BNP is useful for predicting the early development of AKI after LDLT.

## Introduction

Patients who undergo liver transplantation (LT) frequently suffer from acute kidney injury (AKI) after surgery, and the incidence of postoperative AKI ranges from 3.97% to 52%. Severe AKI requires postoperative renal replacement therapy (RRT). Early development of AKI results in chronically impaired kidney function, and this complication is associated with poor patient and graft outcomes [1–4]. Because it takes time for the partial liver graft to sufficiently regenerate in patients who undergo living donor liver transplantation (LDLT), a hyperdynamic condition and portal hypertension subsequently develop during the early postoperative period; these may contribute to early post-transplant AKI [5, 6].

Brain natriuretic peptide (BNP) is primarily secreted by cardiomyocytes in the ventricles under volume or pressure overload conditions [7, 8]. Serum levels of BNP are inversely associated with the glomerular filtration rate (GFR), and BNP concentrations increase in response to a decrease in GFR [9]. BNP levels are higher in patients with AKI than in those without it, and this peptide has increasingly been accepted as a predictor of AKI development [7, 10–12]. However, no studies have described the association between BNP levels and the development of AKI in the LDLT setting. Because patients with end-stage liver disease (ESLD) frequently experience early AKI after LDLT [5, 6], identifying early predictors for post-transplant AKI is required.

We investigated the role of intraoperative serum levels of BNP in predicting the development of early AKI in patients who had undergone LDLT. In addition, we compared intraoperative changes in BNP levels between non-AKI and AKI groups, and investigated BNP levels according to AKI severity stage.

## Patients and methods

### Study population

The study cohort was 328 adult patients (≥19 years old) scheduled for LDLT between January 2011 and December 2017 at our hospital. Only adult patients who underwent elective LDLT were included in this study. Patients who preoperatively suffered from acute or chronic kidney injury, hepatorenal syndrome, or heart disease were excluded. Heart disease was diagnosed by experienced cardiologists, and was defined as having a regional wall motion abnormality with low ejection fraction (<55%), asymmetric septal hypertrophy (septal/free wall ratio ≥ 1:3), and/or atrial fibrillation with a rapid ventricular response (>100 beats/min) [13–15]. The perioperative data of recipients and donors were retrospectively reviewed using the hospital’s electronic medical records system. This study was approved by the Institutional Review Board of our hospital Ethics Committee (KC18RESI0205), and the need for informed consent was waived due to the retrospective study design.

### Perioperative patient management

Our hospital LDLT surgical technique and perioperative patient management protocol have been explained in detail [16]. Briefly, the right lobe from the donor was transplanted into the recipient using the piggyback surgical technique and the middle hepatic vein was reconstructed. After anastomoses of the hepatic vessels, patency of hepatic circulatory inflow and outflow was confirmed by transplant surgeons using Doppler ultrasonography. Intensive and balanced anesthesia management was performed by experienced anesthesiologists under invasive hemodynamic monitoring with serial laboratory measurements.

A triple-drug regimen, including tacrolimus, mycophenolate mofetil, and prednisolone, was used for immunosuppression after LDLT. Tacrolimus was administered to maintain trough levels of 7–10 ng/mL for 1 month after surgery and then gradually tapered to 5–7 ng/mL. Prednisolone was gradually tapered over 1 month after surgery and then discontinued. The dose of mycophenolate mofetil was gradually decreased between 3 and 6 months after surgery. The interleukin-2 receptor antagonist, basiliximab, was administered on the day of surgery and on postoperative day 4.

During the first week, serum creatinine level was measured to detect the early occurrence of AKI. If liver graft function recovered appropriately, tacrolimus administration could be reduced or discontinued in patients with postoperative AKI, and the drug could be replaced by mycophenolate mofetil, prednisolone, or everolimus (mammalian target of rapamycin inhibitor) [17].

### BNP measurement

BNP levels during surgery were measured in the preanhepatic phase, immediately after inducing anesthesia; in the anhepatic phase, starting at the portal venous anastomosis; and in the neohepatic phase, starting at peritoneal closure [18]. Venous blood samples (3 mL) were extracted without stasis into evacuated test tubes (BD Vacutainer, K2 EDTA; Becton, Dickinson and Company, Franklin Lakes, NJ, USA), and BNP levels were analyzed via enzyme-linked immunosorbent assay using a Siemens ADVIA Centaur (Siemens Healthcare Diagnostic, Erlangen, Germany). The analytical process was a fully automated two-site sandwich immunoassay that used direct chemiluminescent technology. The detection range was 2–5,000 pg/mL according to the manufacturer. BNP levels were divided into high *vs*. low based on a cut-off value of 100 pg/mL [19, 20].

### Definition of postoperative AKI

Early AKI after LDLT was determined according to the Kidney Disease: Improving Global Outcomes (KDIGO) criteria [21]. The maximal serum levels of creatinine (sCr) were measured during the first week after surgery and compared to baseline levels before surgery. AKI was defined as follows: increase in sCr ≥ 0.3 mg/dL (≥26.5 μmol/L) by postoperative day (POD) 2 or an increase in sCr ≥ 1.5 times the baseline by POD 7.

AKI severity was classified as follows: AKI stage 1 was a sCr level 1.5–1.9 times the baseline or an increase of ≥0.3 mg/dL (≥26.5 μmol/L) compared to baseline; AKI stage 2 was a sCr level 2.0–2.9 times baseline, and AKI stage 3 was a sCr level 3.0 times the baseline or an increase of ≥ 4.0 mg/dL (≥353.6 μmol/L) or renal replacement therapy (RRT).

The patients were classified into a non-AKI or AKI group based on the development of postoperative AKI.

### Preoperative clinical findings in recipients and donors

The preoperative recipient findings included age, sex, body mass index (BMI), etiology of ESLD, comorbid diseases, previous abdominal surgery, model for end-stage liver disease (MELD) score, hepatic decompensation, transthoracic echocardiography, and laboratory values. Preoperative donor findings included age, sex, BMI, and comorbid diseases.

Intraoperative findings included operation time; severe post-reperfusion syndrome (defined as blood pressure and/or heart rate > 30% of the anhepatic values and continuing for more than 5 min; hemodynamically instable arrhythmia, such as severe bradycardia [<40 beats/min] with irregularity; and requiring rescue vasopressors, such as an intravenous epinephrine bolus [≥100 μg] or continuous infusion of norepinephrine [≥0.1 μg/kg/min] during the neohepatic period) [22]; vital sign parameters, including central venous pressure (CVP), mean pulmonary arterial pressure (MPAP), stroke volume variation (SVV), cardiac index (CI), systemic vascular resistive index (SVRI), mean blood pressure (MBP), and heart rate (HR); laboratory values; blood product transfusions; hourly fluid administration; hourly urine output; and total number of drugs administered.

Liver graft findings included total duration of ischemia, steatotic percentage, graft-recipient-weight ratio, graft to standard liver volume ratio, average portal venous flow, and the hepatic artery resistive index during POD 5.

### Statistical analyses

Continuous data were compared using the Mann–Whitney *U*-test and are expressed as medians and interquartile ranges. The normality of the continuous data was identified using the Shapiro–Wilk test. Categorical data were evaluated using the χ^2^ test or Fisher’s exact test, as appropriate, and are expressed as numbers and proportions. The linear-by-linear association test was used to analyze trends in BNP levels according to KDIGO stage. Intraoperative changes in BNP levels were analyzed by repeated-measures analysis of variance and the Bonferroni’s *post hoc* method. BNP levels were compared between the preanhepatic and neohepatic phases using the Wilcoxon singed-rank test. BNP levels were evaluated according to KDIGO stage using the Kruskal–Wallis test and Bonferroni’s *post hoc* method. After dividing serum levels of BNP into low vs. high based on a cut-off value of 100 pg/mL, intraoperative changes in the proportions of patients with high BNP levels were analyzed using Cochran’s Q test with the McNemar *post hoc* test. The associations between perioperative recipient and donor-graft variables and the development of AKI were evaluated using univariate and multivariate logistic regression analyses. Potentially significant factors (*p*≤0.1) in univariate analyses were entered into multivariate forward and backward regression analyses. The values are presented with odds ratios and 95% confidence intervals (CIs). When multiple perioperative factors were inter-correlated, the most clinically important factors were selected. The predictive accuracies of independent factors for AKI were evaluated using the area under the receiver operating characteristic curve (AUC). All tests were two-sided, and a *p*<0.05 was taken to indicate significance. Statistical analyses were performed using SPSS (ver. 22.0 for Windows; SPSS Inc., Chicago, IL, USA) and MEDCALC (ver. 11.0 for Windows; MedCalc Software, Mariakerke, Belgium).

## Results

Fifty-six patients were excluded from this study because of a preoperative history of acute or chronic kidney injury (n = 15), hepatorenal syndrome (n = 5), heart disease (n = 8), emergency LDLT (n = 18), or missing laboratory variables (n = 10). Therefore, 272 patients were analyzed. The study cohort was mainly male (69.1%), the median age was 54 (49–59) years, and the median BMI was 24.3 (22.2–27.2) kg/m^2^. The etiologies for LDLT were hepatitis B (54.8%), alcoholic hepatitis (17.3%), hepatitis C (11.0%), autoimmune hepatitis (2.9%), drug and toxic hepatitis (2.2%), hepatitis A (1.8%), and cryptogenic hepatitis (9.9%). The median MELD score was 14 (7–25) points. According to the KDIGO criteria, 61 patients (22.4%) suffered from AKI immediately after LDLT. Severity by AKI stage was as follows: 28 patients in stage 1 (10.3%), 18 patients in stage 2 (6.6%), and 15 patients in stage 3 (5.5%).

Patients with AKI had a higher preoperative BMI and a greater incidence of ascites (>1 L) than those without AKI (Table 1). Levels of albumin and calcium in the blood were lower in the AKI group than in the non-AKI group; however, total bilirubin levels were higher in the AKI group than in the non-AKI group. No differences in preoperative donor findings were observed between the non-AKI and AKI groups (Table 1).

**Table 1:** Comparisons of preoperative recipient and donor findings between non-AKI and AKI groups in patients undergoing living donor liver transplantation.

Group	non-AKI	AKI	
n	211	61	*p*
**Recipient finding**			
Age (years)	54 (49–59)	53 (48–60)	0.824
Sex (male)	142 (67.3%)	46 (75.4%)	0.227
Body mass index (kg/m^2^)	24.0 (22.1–27.1)	25.5 (23.4–27.9)	0.033
Etiology			0.801
Alcohol	33 (15.6%)	14 (23.0%)	
Hepatitis A	4 (1.9%)	1 (1.6%)	
Hepatitis B	116 (55.0%)	33 (54.1%)	
Hepatitis C	26 (12.3%)	4 (6.6%)	
Autoimmune	6 (2.8%)	2 (3.3%)	
Drug & Toxin	5 (2.4%)	1 (1.6%)	
Cryptogenic	21 (10.0%)	6 (9.8%)	
Comorbidity			
Diabetes mellitus	50 (23.7%)	20 (32.8%)	0.153
Hypertension	44 (20.9%)	15 (24.6%)	0.533
Lung disease	32 (15.2%)	6 (9.8%)	0.290
History of abdomen surgery	49 (23.2%)	15 (24.6%)	0.825
MELD score (points)	13 (6–25)	17 (9–25)	0.437
Hepatic decompensation			
Encephalopathy (West-Haven grade III or IV)	20 (9.6%)	5 (8.2%)	0.745
Varix	56 (26.5%)	19 (31.1%)	0.478
Ascites (>1L)	92 (43.6%)	37 (60.7%)	0.019
Transthoracic echocardiography			
Ejection fraction (%)	65 (62–66)	65 (62–67)	0.243
Diastolic dysfunction (Grade II or III)	59 (28.0%)	17 (27.9%)	0.989
Laboratory findings			
Hematocrit (%)	29 (25–34)	28 (25–31)	0.151
White blood cell count (x 10^9^/L)	4.3 (2.6–6.5)	4.6 (3.2–7.7)	0.374
Neutrophil (%)	61.7 (51.8–75.0)	62.0 (50.2–73.8)	0.908
Lymphocyte (%)	22.4 (13.0–32.3)	19.0 (12.5–31.8)	0.668
C-reactive protein (mg/dL)	0.4 (0.1–1.7)	0.8 (0.2–2.1)	0.070
Platelet count (x 10^9^/L)	62.0 (45.0–100.0)	58.0 (41.5–74.5)	0.198
International normalized ratio	1.5 (1.2–2.1)	1.7 (1.4–2.2)	0.106
Glucose (mg/dL)	112 (93–141)	118 (100–146)	0.409
Creatinine (mg/dL)	0.9 (0.7–1.3)	0.9 (0.7–1.4)	0.964
Albumin (g/dL)	3.1 (2.7–3.6)	2.8 (2.5–3.3)	0.002
Aspartate aminotransferase (U/L)	47 (30–92)	51 (39–90)	0.355
Alanine aminotransferase (U/L)	30 (19–62)	31 (23–70)	0.362
Total bilirubin (mg/dL)	2.1 (0.9–13.5)	4.7 (1.6–18.9)	0.029
Sodium (mEq/L)	140.0 (136.0–142.0)	138 (134–142)	0.065
Calcium (mg/dL)	8.4 (8.0–8.8)	8.2 (7.8–8.6)	0.047
Potassium (mEq/L)	3.9 (3.6–4.3)	3.9 (3.6–4.3)	0.989
Ammonia (mcg/dL)	96.0 (64.0–154.0)	93.0 (66.0–139.5)	0.930
**Donor finding**			
Age (years)	36 (26–43)	36 (30–49)	0.118
Gender (Male)	149 (70.6%)	42 (68.9%)	0.791
Body mass index (kg/m^2^)	23.7 (22.1–25.5)	23.7 (23.0–23.7)	0.552
Diabetes mellitus	2 (0.9%)	0 (0.0%)	1.000
Hypertension	3 (1.4%)	1 (1.6%)	1.000

**Abbreviaion:** AKI, acute kidney injury; MELD, model for end-stage liver disease

**NOTE:** Values are expressed as median (interquartile) and number (proportion).

Average levels of hemoglobin and platelet counts were intraoperatively lower in the AKI group than in the non-AKI group; however, the levels of BNP and potassium were higher in the AKI group (Table 2). Patients with AKI were transfused with more packed red blood cells (PRBCs), fresh frozen plasma (FFP), or cryoprecipitate than those without AKI. Hourly urine output was lower in the AKI group. No differences in liver graft findings were detected between the groups (Table 2).

**Table 2:** Comparisons of intraoperative recipient and graft findings between non-AKI and AKI groups in patients undergoing living donor liver transplantation.

Group	non-AKI	AKI	
n	211	61	*p*
**Recipient finding**			
Operation time (min)	495 (450–545)	500 (430–580)	0.568
Severe postreperfusion syndrome	46 (21.8%)	19 (31.1%)	0.132
Average of vital signs during surgery			
Central venous pressure (mmHg)	9 (8–12)	9 (7–11)	0.230
MPAP (mmHg)	23 (20–25)	23 (19–25)	0.684
Stroke volume variation (%)	7 (5–9)	7 (5–8)	0.461
Cardiac index (L/min/m^2^)	4.2 (3.6–4.6)	4.2 (3.5–4.4)	0.264
SVRI (dynes-sec/cm^-5^/m^2^)	1317 (1088–1476)	1317 (1173–1429)	0.744
Mean blood pressure (mmHg)	78 (70–86)	77 (71–87)	0.735
Heart rate (beats/min)	90 (80–101)	88 (80–98)	0.283
Average of laboratory values during surgery			
Brain natriuretic peptide (pg/mL)	92 (44–167)	120 (71–172)	0.026
Hemoglobin (g/dL)	9.8 (8.8–10.3)	9.2 (8.5–9.8)	0.004
White blood cell count (x 10^9^/L)	6.8 (5.2–9.3)	7.0 (6.0–9.0)	0.598
Neutrophil (%)	82.8 (78.5–87.4)	81.3 (78.7–84.8)	0.143
Lymphocyte (%)	35.0 (33.4–37.6)	35.4 (33.8–38.4)	0.124
Potassium (mEq/L)	3.9 (3.5–4.2)	4.1 (3.6–4.5)	0.031
aPTT (sec)	67.9 (52.7–85.2)	74.5 (62.2–90.7)	0.060
International normalized ratio	2.0 (1.6–2.0)	2.0 (1.8–2.3)	0.044
Platelet count (x 10^9^/L)	73.7 (55.6–82.0)	63.3 (52.8–74.1)	0.033
Fibrinogen (mg/dL)	122 (100–140)	118 (91–131)	0.237
Lactate (mmol/L)	4.1 (3.7–4.8)	4.1 (3.5–4.7)	0.371
Glucose (mg/dL)	163 (144–180)	153 (137–175)	0.279
Total amount of blood product transfusion (unit)			
Packed red blood cell	7 (4–11)	10 (5–16)	0.003
Fresh frozen plasma	6 (4–10)	8 (5–12)	0.030
Single donor platelet	0 (0–1)	0 (0–1)	0.763
Cryoprecipitate	0 (0–0)	0 (0–0)	0.009
Hourly fluid administration (mL/kg/h)	11.6 (9.3–14.8)	12.6 (8.8–16.5)	0.388
Hourly urine output (mL/kg/h)	1.1 (0.5–2.0)	0.7 (0.5–1.6)	0.024
Total amount of drug administration			
CaCl_2_ (mg)	279 (0–625)	365 (0–825)	0.653
Bicarbonate (mEq)	0 (0–50)	0 (0–80)	0.747
Insulin (unit)	6 (2–15)	10 (0–18)	0.286
Furosemide (mg)	5 (0–20)	10 (0–20)	0.873
**Liver graft finding**			
Total graft ischemic time (min)	92 (68–125)	109 (80–129)	0.053
Steatotic percentage (%)	5.0 (0.0–5.6)	5.0 (1.0–5.6)	0.179
Graft-recipient-weight-ratio (%)	1.2 (1.1–1.6)	1.2 (1.0–1.5)	0.225
Graft to standard liver volume ratio (%)	58.2 (51.2–69.6)	55.2 (48.3–66.5)	0.128
Average of hepatic circulation during postoperative day 5			
Portal venous flow (mL/min)	1991.0 (1715.5–2298.0)	2123.9 (1756.3–2423.7)	0.481
Hepatic artery resistive index	0.7 (0.6–0.8)	0.7 (0.6–0.8)	0.428

**Abbreviations**: AKI, acute kidney injury; MPAP, mean pulmonary arterial pressure; SVRI, systemic vascular resistive index; aPTT, activated partial thrombin time

**NOTE:** Values are expressed as median (interquartile) and number (proportion).

BNP levels in the neohepatic phase were higher in the AKI group than in the non-AKI group; however, levels in the preanhepatic and anhepatic phases were comparable between the two groups (Table 3). BNP levels significantly decreased from the preanhepatic phase to the neohepatic phase in both groups. However, the decreases were similar between the two groups: –20 (–75 to 3) *vs*. –27 (–80 to 25) pg/mL in the AKI group (*p* = 0.856).

**Table 3:** Changes in serum levels of brain natriuretic peptide between non-acute kidney injury and acute kidney injury groups during living donor liver transplantation.

Group	non-AKI	AKI	
n	211	61	*p*
Brain natriuretic peptide (pg/mL)			
Preanhepatic phase	102 (51–203)	146 (66–195)	0.082
Anhepatic phase	84 (38–121)	111 (46–127)	0.390
Neohepatic phase	89 (41–135) ^#^	115 (71–242) ^#^	<0.001

**Abbreviation:** AKI, acute kidney injury

**NOTE:** Values are expressed as median and interquartile.

^#^*p* < 0.05 between the preanhepatic and neohepatic phases

BNP levels increased with worsening AKI severity compared to levels in the neohepatic phase (Table 4). Patients with AKI stage 3 had significantly higher BNP levels than the other groups. BNP levels significantly decreased from the preanhepatic phase to the neohepatic phase in patients with only AKI stage 1. Median (interquartile) changes from the preanhepatic phase to the neohepatic phase were as follows: –70 (–114 to –19) pg/mL in AKI stage 1; –27 (–66 to 47) pg/mL in AKI stage 2; and 90 (–76 to 100) pg/mL in AKI stage 3.

**Table 4:** Comparisons of serum brain natriuretic peptide levels according to KDIGO stage during living donor liver transplantation.

KDIGO stage	Stage 1	Stage 2	Stage 3	*p*
n	28	18	15	
Brain natriuretic peptide (pg/mL)				
Preanhepatic phase	152 (96–196)	145 (48–183)	94 (44–408)	0.744
Anhepatic phase	82 (51–146)	114 (39–117)	111 (39–116)	0.970
Neohepatic phase	107 (60–131) ^#^	108 (68–172)	192 (130–327)*	0.002

**Abbreviation:** KDIGO, Kidney Disease: Improving Global Outcomes

**NOTE**: Values are expressed as median and interquartile.

*p≤0.01 between the stage 3 *vs*. stage 1 or 2

^#^*p*<0.001 between the preanhepatic and neohepatic phases

Based on BNP levels ≥ 100 pg/mL, the incidence rate of elevated levels was higher in the AKI group than in the non-AKI group in the neohepatic phase (Table 5).

**Table 5:** Incidences of high serum levels of brain natriuretic peptide (≥100 pg/mL) between non-acute kidney injury and acute kidney injury groups during living donor liver transplantation.

Group	non-AKI	AKI	
n	211	61	*p*
Brain natriuretic peptide			
Preanehpatic phase			
<100 pg/mL	105 (49.8%)	22 (36.1%)	0.059
≥100 pg/mL	106 (50.2%)	39 (63.9%)	
Anhepatic phase			
<100 pg/mL	114 (54.0%)	30 (49.2%)	0.504
≥100 pg/mL	97 (46.0%)	31 (50.8%)	
Neohepatic phase			
<100 pg/mL	113 (53.6%)	18 (29.5%)	0.001
≥100 pg/mL	98 (46.4%)	43 (70.5%)	

**Abbreviation:** AKI, acute kidney injury

**NOTE:** Values are expressed as numbers and proportions

After dichotomizing BNP levels at a cut-off value of 100 pg/mL, the proportions of patients with high levels were higher in the stage 3 group than in the other groups in the neohepatic phase (Table 6). The proportion of patients with high levels significantly increased from the preanhepatic to the neohepatic phase in AKT stage 3.

**Table 6:** Incidences of high serum levels of brain natriuretic peptide (≥100 pg/mL) according to KDIGO stage during living donor liver transplantation.

KDIGO stage	Stage 1	Stage 2	Stage 3	
n	28	18	15	*p*
Brain natriuretic peptide				
Preanehpatic phase				0.262
<100 pg/mL	8 (28.6%)	6 (33.3%)	8 (53.3%)	
≥100 pg/mL	20 (71.4%)	12 (66.7%)	7 (46.7%)	
Anhepatic phase				0.812
<100 pg/mL	15 (53.6%)	8 (44.4%)	7 (46.7%)	
≥100 pg/mL	13 (46.4%)	10 (55.6%)	8 (53.3%)	
Neohepatic phase				0.012
<100 pg/mL	12 (42.9%)	6 (33.3%)	0 (0.0%)	
≥100 pg/mL	16 (57.1%)	12 (66.7%)	15 (100.0%)^#^	

**Abbreviation:** KDIGO, Kidney Disease: Improving Global Outcomes

**NOTE**: Values are expressed as numbers and proportions.

^#^*p*<0.01 between the preanehpatic and neohepatic phases

The associations between the perioperative factors and early AKI development after LDLT are shown in Table 7. In univariate analyses, preoperative recipient factors, intraoperative recipient factors, and donor and graft factors were potentially significant. In multivariate analyses, BNP levels in the neohepatic phase as well as the total amount of PRBC transfusion and total duration of graft ischemia were significantly associated with early AKI development after LDLT.

**Table 7:** Associations between perioperative factors and early acute kidney injury development after living donor liver transplantation.

	Univariate logistic regression				Multivariate logistic regression			
	*β*	OR	95% CI	*p*	*β*	OR	95% CI	*p*
**Preoperative recipient factor**								
Model for end-stage liver disease (point)	0.003	1.003	0.979–1.028	0.809				
Body mass index (kg/m^2^)	0.068	1.070	1.003–1.142	0.042				
Ascites (>1L)	0.690	1.994	1.115–3.566	0.020				
Albumin (g/dL)	-0.434	0.648	0.409–1.027	0.065				
Sodium (mEq/L)	-0.044	0.957	0.911–1.006	0.084				
**Intraoperative recipient factor**								
Serum brain natriuretic peptide level at the neohepatic phase (pg/mL)	0.004	1.004	1.001–1.006	0.002	0.004	1.004	1.001–1.006	0.002
Average of laboratory values during surgery								
Hemoglobin (g/dL)	-0.312	0.732	0.578–0.927	0.009				
Potassium (mEq/L)	0.689	1.992	1.125–3.528	0.018				
aPTT (sec)	0.012	1.012	1.000–1.025	0.056				
International normalized ratio	0.404	1.498	0.976–2.298	0.064				
Platelet count (x 10^9^/L)	-0.009	0.991	0.981–1.001	0.083				
Fibrinogen (mg/dL)	-0.006	0.994	0.987–1.001	0.093				
Total amount of blood product transfusion (unit)								
Packed red blood cell	0.048	1.049	1.014–1.084	0.005	0.040	1.041	1.006–1.077	0.020
Fresh frozen plasma	0.060	1.062	1.014–1.112	0.011				
Cryoprecipitate	0.128	1.137	1.034–1.250	0.008				
Hourly urine output (mL/kg/h)	-0.352	0.703	0.513–0.964	0.029				
**Donor & graft factors**								
Total graft ischemic time (min)	0.004	1.004	1.001–1.007	0.014	0.015	1.004	1.001–1.007	0.015

**Abbreviations:** OR, Odds ratio; CI, confidence interval; aPTT, activated partial thrombin time

This model showed moderate accuracy for predicting early AKI development after LDLT (AUC = 0.69; 95% CI = 0.61–0.76; *p*<0.001). S1 Table shows the cut-off levels of independent factors, including BNP levels in the neohepatic phase, total amount of PRBC transfusion during surgery, and total ischemic time of liver graft, for postoperative AKI development in LDLT patients.

## Discussion

In patients undergoing major surgery, elevated levels of BNP can be used to identify patients at high risk for all-cause mortality, including cardiac-origin death, and major cardiovascular events [23]. Regarding to its diagnostic accuracy for cardiac dysfunction, serum levels of BNP ≥ 100 pg/mL are strongly and independently associated with congestive heart failure [8, 19, 20]; this level may also be associated with the clinically accepted cut-off level for high *vs*. low BNP levels in an LT setting. The associations between natriuretic peptides and AKI development have been investigated clinically. Cardiac biomarkers, including N-terminal pro-BNP, are strongly associated with the occurrence of AKI and are increasingly becoming a requirement for RRT in clinically ill patients without cardiac disease in the intensive care unit (ICU) [12]. In patients with complicated ST-segment elevation myocardial infarction, higher BNP levels are associated with the development and severity of AKI [24]. The association between heart failure and kidney dysfunction has been established as cardio-renal syndrome, and natriuretic peptide is considered a predictive biomarker for kidney injury [7]. Preoperative BNP levels are a strong and independent biomarker of hemodynamic strain and AKI development, and can help improve AKI risk stratification in patients at high risk for AKI before cardiac surgery [11]. However, the mechanism underlying the relation between high BNP levels and AKI development remains unclear. One potential explanation is that cardiac dysfunction, such as high right arterial pressure and low cardiac output, may impair renal blood flow and cause venous congestion in the kidney [25]. There are close associations among ventricular dilation, high venous pressure, and kidney dysfunction, such that increased venous pressure may lead to backward pressure throughout the venous circulation, including the veins of the kidney [26, 27]. This pathological condition may result in renal congestion and decrease GFR and sodium excretion [28, 29]. Such decreases in elevated venous congestion before or during cardiac surgery are considered important kidney-sparing treatment strategies, and serum levels of BNP could play a role in assessing the efficacy of perioperative attenuation of hemodynamic strain related to volume expansion or pressure overload [25, 30, 31].

In patients undergoing LT, high preoperative BNP levels are a significant predictor of patient mortality and morbidity, such as higher rates of RRT requirement [32]. In LDLT, patients with early allograft dysfunction (EAD) have higher BNP levels during surgery than those without EAD [18]. However, in our study, unlike previous studies in major surgery settings [11, 12, 32, 33], the differences in serum levels of BNP between the non-AKI and AKI groups occurred as the surgery proceeded. Although BNP levels in the AKI group were higher than those in the non-AKI group in the preanhepatic phase, the difference was not statistically significant. In the neohepatic phase, the difference in BNP levels between the two groups was significant, and the proportion of patients with high levels (≥100 pg/mL) was higher in the AKI group than in the non-AKI group. During LDLT, BNP levels decreased to a similar extent in both the non-AKI and AKI groups. However, according to AKI stage, BNP levels only significantly decreased in the AKI stage 1 group from the preanehpatic phase to the neohepatic phase. In particular, the levels of all AKI stage 3 patients increased to more than 100 pg/mL, and the proportion of patients with high levels also significantly increased through the surgical phases in that group. Therefore, our findings suggest that BNP levels are related to the development of AKI in patients undergoing LDLT; and as the severity of AKI increases, the levels do not decrease during surgery but rather remain high until the neohepatic phase. These findings are in line with previous studies [34, 35] indicating that B-type natriuretic peptide is strongly associated with the worst stage of AKI requiring RRT, and that BNP levels show a significant gradient depending on the severity of AKI. However, we could not account for AKI-related factors affecting the intraoperative changes in BNP levels. Further research is needed to investigate the roles of elevated BNP levels in the development of primary or secondary AKI and to determine the optimal cut-off level of BNP for AKI after LDLT. Our findings suggest that monitoring BNP levels in each surgical phase will help identify patients at high risk for the development of AKI after LDLT.

In our study, PRBC transfusion was a major risk factor for AKI development in patients who had undergone LT. A previous study suggested that PRBC transfusion < 6 units is associated with a decrease in the development of AKI in LT patients [36]. In other studies [2, 3], patients who suffered from AKI after LT were transfused with more blood products, including PRBCs, than those without AKI. The potential mechanisms for the noxious effects of PRBC transfusion on kidney function are an increased inflammatory response, reduced oxygen delivery, and aggravated tissue oxidative stress. These risk factors are affected by the volume of transfusions, the duration of preservation of the blood products, and the patient’s vulnerability [37]. We found that intraoperative PRBC transfusion was independently associated with AKI development after surgery, in line with previous studies. Particularly, anemic patients are more vulnerable to the development of AKI because native red blood cells defend against oxidative injury and a PRBC transfusion aggravates oxidative stress [38]. Because patients who undergo LDLT are usually preoperatively anemic [39], lower oxygen content immediately after graft reperfusion is associated with AKI development [16]; thus, preoperative patient buildup to reduce anemia, such as intravenous iron supplementation [40], and meticulous intraoperative PRBC transfusions may be required to prevent the early occurrence of AKI after surgery.

Prolonged graft ischemia duration was associated with the development of AKI after LT, which may be associated with graft ischemia-reperfusion injury [4]. One study demonstrated that warm ischemic time (WIT) is a strong and independent factor of postoperative continuing kidney impairment in patients who suffer from severe subacute kidney injury [41]. Increasing WIT is an important factor that significantly affects the development of AKI in patients who undergo LT [42, 43]. Another study suggested that an AKI group had longer duration cold and warm ischemic times than a non-AKI group, and cold and warm ischemic times were independent risk factors for AKI development in LT [1]. When a graft is damaged during a long ischemic time, this may contribute to a greater production of reactive oxygen molecules (i.e., superoxide anions, hydrogen peroxide, and hydroxyl radicals) in the graft, leading to kidney cell injury [44]. Although LDLT reportedly has a shorter graft ischemia duration than deceased donor LT [45], our study suggests that total ischemia time was an independent factor for AKI development after surgery. Because graft ischemia duration may be a modifiable factor affected by surgical technique or graft allocation, efforts to reduce this are likely to decrease the incidence of AKI after LDLT. Many studies in an LT setting have used regional perfusion devices to reduce ischemia-reperfusion injury during graft storage and to extend graft storage duration, which have shown positive outcomes [46, 47].

Some limitations of this study should be discussed. First, sCr is the most useful marker for estimating the degree of kidney function [48, 49]. However, they are affected by nutrition, exercise, stress, age, pregnancy, and skeletal muscle mass [50]. Particularly, patients with ESLD show decreased sCr levels due to reduced Cr synthesis in the liver and skeletal muscles, and elevated tubular Cr secretion. Cr-based estimates of GFR may be less accurate for predicting AKI in patients with ESLD [51]. Second, there were small numbers of patients in each AKI stage (e.g., only 15 in the AKI stage 3 group) and thus our study was underpowered to reliably predict AKI development after LDLT. Therefore, we could not suggest optimal cut-off values for serum levels of BNP, units of PRBC transfusion, and total graft ischemic time. However, the strength of our study is suggesting that BNP levels during surgery could serve as a readily available and useful factor for identifying LDLT patients at high risk for AKI development. In addition, because this study was conducted retrospectively at a single tertiary center, some clinical variables were unavoidably missed during data collection, and confounding factors could not be excluded during data analyses. A further randomized control study would be helpful to overcome selection bias.

In conclusion, serum levels of BNP are a useful biomarker for predicting the development of early AKI in patients following LDLT. We found that, when BNP levels markedly decreased during surgery, postoperative kidney function was maintained within the normal range. However, patients with moderate AKI did not show significant decreases in BNP levels during surgery, and patients with severe AKI had continuously high levels (≥100 pg/mL). Therefore, monitoring intraoperative changes in BNP levels could be helpful to identify patients at high risk for postoperative kidney injury. Further studies are required to investigate the critical factors affecting BNP levels during LDLT. In addition, careful intraoperative transfusion of PRBCs and endeavors to reduce graft ischemia duration would be helpful to maintain kidney function after LDLT.

## Supporting information

S1 Table Cut-off values of serum levels of brain natriuretic peptide (BNP) in the neohepatic phase, total amount of packed red blood cell (PRBC) transfusion during surgery, and total ischemic time of liver graft for early acute kidney injury development in living donor liver transplantation. (DOCX)

## Abbreviations

LT: liver transplantation
AKI: acute kidney injury
RRT: renal replacement therapy
LDLT: living donor liver transplantation
BNP: brain natriuretic peptide
GFR: glomerular filtration rate
ESLD: end-stage liver disease
KDIGO: the Kidney Disease: Improving Global Outcomes
sCr: serum creatinine
BMI: body mass index
MELD: model for end-stage liver disease
CVP: central venous pressure
MPAP: mean pulmonary arterial pressure
SVV: stroke volume variation
PRBC: packed red blood cell
FFP: fresh frozen plasma
