# Supplementary material for: Role of serum levels of intraoperative brain natriuretic peptide for predicting acute kidney injury in living donor liver transplantation
Source: PLoS One. 2018 Dec 17;13(12):e0209164. doi: 10.1371/journal.pone.0209164 (PMC6296541; doi:10.1371/journal.pone.0209164)
Supplement: S1 Table — (DOCX) [file pone.0209164.s001.docx]

**Supporting information**

| **S1 Table.** Cut-off values of serum levels of brain natriuretic peptide (BNP) in the neohepatic phase, total amount of packed red blood cell (PRBC) transfusion during surgery, and total ischemic time of liver graft for early acute kidney injury development in living donor liver transplantation | | | | | | |
| --- | --- | --- | --- | --- | --- | --- |
|  | **Cut-off level** | **AUC** | **95% CI** | **Sensitivity** | **Specificity** | ***p*** |
| **BNP** | 102 pg/mL | 0.65 | 0.59 - 0.71 | 70.5% | 55.0% | <0.001 |
| **PRBC** | 8 unit | 0.62 | 0.56 - 0.68 | 62.3% | 63.0% | <0.01 |
| **Total ischemic time** | 85 minute | 0.59 | 0.52 - 0.64 | 73.8% | 45.5% | <0.05 |
| **Abbreviation:** AUC, area under the receiver operating characteristic curve; CI, confidence interval | | | | | | |
